# Fat Mass- and Obesity-Associated Protein (FTO) Promotes the Proliferation of Goat Skeletal Muscle Satellite Cells by Stabilizing DAG1 mRNA in an IGF2BP1-Related m^6^A Manner

**DOI:** 10.3390/ijms25189804

**Published:** 2024-09-11

**Authors:** Jiangzhen Yao, Liang Xu, Zihao Zhao, Dinghui Dai, Siyuan Zhan, Jiaxue Cao, Jiazhong Guo, Tao Zhong, Linjie Wang, Li Li, Hongping Zhang

**Affiliations:** 1 Farm Animal Genetic Resources Exploration and Innovation Key Laboratory of Sichuan Province, Sichuan Agricultural University, Chengdu 611130, China; 18503449958@163.com (J.Y.); xl18383535205@163.com (L.X.); z15528133630@163.com (Z.Z.); 71317@sicau.edu.cn (D.D.); siyuanzhan@sicau.edu.cn (S.Z.); jiaxuecao@sicau.edu.cn (J.C.); jiazhong.guo@sicau.edu.cn (J.G.); zhongtao@sicau.edu.cn (T.Z.); wanglinjie@sicau.edu.cn (L.W.); 2 Key Laboratory of Livestock and Poultry Multi-Omics, Ministry of Agriculture and Rural Affairs, College of Animal and Technology, Sichuan Agricultural University, Chengdu 611130, China

**Keywords:** RNA m^6^A methylation, FTO, DAG1, goat, MuSCs

## Abstract

Skeletal muscle development is spotlighted in mammals since it closely relates to animal health and economic benefits to the breeding industry. Researchers have successfully unveiled many regulatory factors and mechanisms involving myogenesis. However, the effect of N^6^-methyladenosine (m^6^A) modification, especially demethylase and its regulated genes, on muscle development remains to be further explored. Here, we found that the typical demethylase FTO (fat mass- and obesity-associated protein) was highly enriched in goats’ longissimus dorsi (LD) muscles. In addition, the level of m^6^A modification on transcripts was negatively regulated by FTO during the proliferation of goat skeletal muscle satellite cells (MuSCs). Moreover, a deficiency of FTO in MuSCs significantly retarded their proliferation and promoted the expression of dystrophin-associated protein 1 (DAG1). m^6^A modifications of *DAG1* mRNA were efficiently altered by FTO. Intriguingly, the results of DAG1 levels and its m^6^A enrichment from FB23-2 (FTO demethylase inhibitor)-treated cells were consistent with those of the FTO knockdown, indicating that the regulation of FTO on DAG1 depended on m^6^A modification. Further experiments showed that interfering FTO improved m^6^A modification at site DAG1-122, recognized by Insulin-like growth factor 2 mRNA-binding protein 1 (IGF2BP1) and consequently stabilized *DAG1* transcripts. Our study suggests that FTO promotes the proliferation of MuSCs by regulating the expression of DAG1 through m^6^A modification. This will extend our knowledge of the m^6^A-related mechanism of skeletal muscle development in animals.

## 1. Introduction

Goat meat is important in agriculture and animal husbandry in many countries [1]. The growth and development process of skeletal muscle includes several steps: progenitor cell/satellite cell generation, myoblast proliferation, directional differentiation, myocyte fusion to form multinuclear muscle tubes, and further formation of mature muscle fibers [2]. Recent studies suggest that in addition to many transcription factors, such as myogenic regulatory factors (MRFs) [3], specific epigenetic mechanisms may play an important role in controlling skeletal muscle transcription patterns [4]. N^6^-methyladenosine (m^6^A) modification is the most common mRNA methylation modification in eukaryotes [5]. The relationship between m^6^A modification and muscle development is complex and may involve multiple genes and regulatory factors. Therefore, it is necessary to explore the role of m^6^A modification on myogenesis further.

m^6^A methyltransferase (writer), demethylase (eraser), and reader protein (reader) participate in the RNA m^6^A methylation modification process. m^6^A writing requires the participation of a variety of proteins to form a methyltransferase complex with methyltransferase-like 3 (METTL3) and methyltransferase-like 14 (METTL14) as the main components [6]. METTL3 acts as a catalyst, and METTL14 stabilizes the structure of the complex, recruiting proteins such as WTAP to recognize catalytic substrates—specific RNA sequence RRACH (R: A, G; A: m^6^A; H: A, C, U) [7].

As a demethylase (eraser), FTO (fat mass- and obesity-associated protein) was discovered in 2011 [8]. In 2013, the second RNA demethylase of the same family was discovered—AlkB homology 5 (ALKBH5) [9]. These findings suggest that m^6^A modification is a dynamic and reversible process. Moreover, reading proteins, including the YTHDF family, YTHDC family, and Insulin-like growth factor 2 mRNA-binding protein 1 (IGF2BP1), recognize the m^6^A sites and consequently alter mRNA processing, such as by affecting the shearing process [10], regulating stability [11], and promoting translation and degradation [12]. As the first demethylase to be discovered [8], FTO is closely related to the proliferation and differentiation of myoblasts. However, its regulatory mechanism on myocyte proliferation and whether it depends on m^6^A modification remains to be further explored.

Here, we explored and found the high enrichment of FTO in goat skeletal muscles and its close association with m^6^A modification on transcripts of goat proliferating muscle satellite cells (MuSCs). Mechanistically, during myocyte proliferation, FTO inhibits dystrophin-associated protein 1 (DAG1) mRNA dependent on m^6^A modification associated with the m^6^A reading protein IGF2BP1. Our results provide a reference for further understanding the relationship between m^6^A modification and skeletal muscle development. 

## 2. Results

### 2.1. FTO Knockdown Suppresses the Proliferation of Goat MuSCs

**Figure 1:**
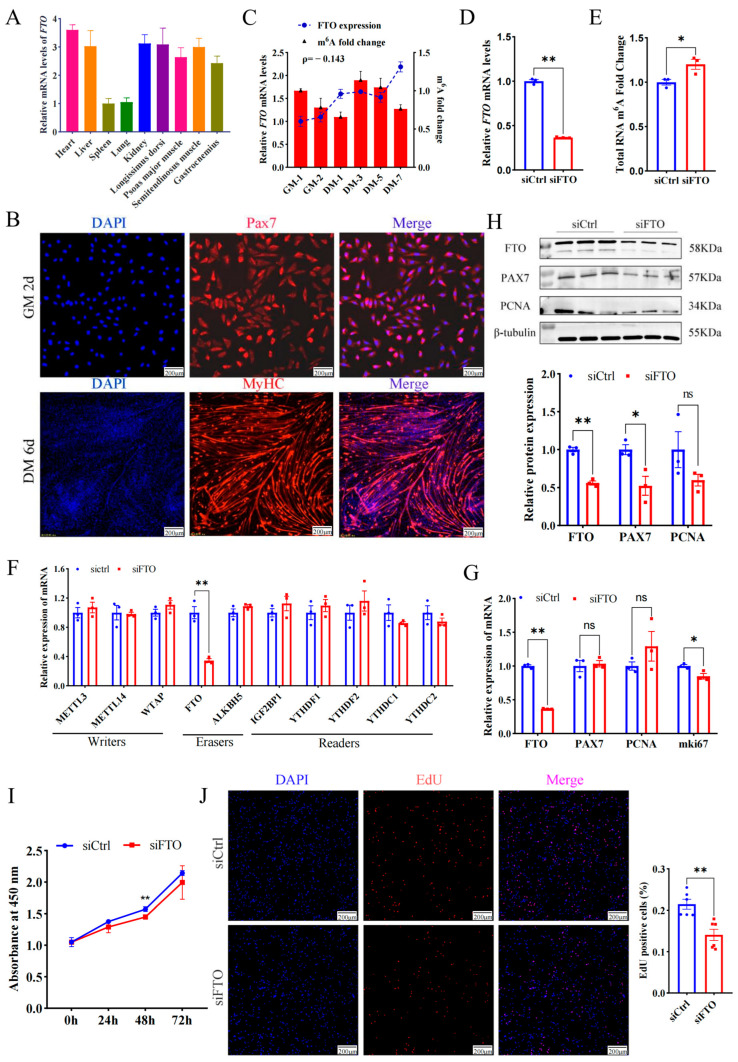
Deficiency of FTO suppresses the proliferation of goat MuSCs. (**A**) Expression of *FTO* in different tissues of goats. (**B**) Cells immunofluorescent stained with anti-PAX7 (MuSCs cultured in growth medium (GM) for 2 days) and anti-MYHC (MuSCs cultured in differentiation medium (DM) for 6 days). Scale bar: 200 μm. (**C**) *FTO* mRNA and m^6^A changes during MuSCs (cultured in the growth medium for 1 and 2 days and differentiation medium for 1, 3, 5, and 7 days). (**D**) mRNA level of *FTO* in cells treated with siFTO. (**E**) The m^6^A of total RNA affected by FTO knockdown. (**F**) Effect of FTO knockdown on gene expression of m^6^A modified enzymes. (**G**) mRNA changes in myoblast proliferation marker genes after FTO knockdown. (**H**) Protein of myoblast proliferation genes affected by deficiency of FTO. (**I**) CCK8 assay of the viability of MuSCs. (**J**) The number of new cells stained with EdU. Scale bar: 200 μm. Results are represented as the mean ± SEM, * *p* < 0.05, ** *p* < 0.01, and ns indicate insignificance.

In addition to the spleen and lung, *FTO* mRNA was highly enriched in the heart, liver, kidney, and four types of skeletal muscles, including the longissimus dorsi muscle (LD), semitendinosus muscle, psoas major muscle, and gastrocnemius muscle (Figure 1A), implying FTO is closely related to muscle development. To profile FTO in the proliferating myoblasts, MuSCs from newborn goat LD muscles were isolated, purified, and identified (Figure 1B). FTO gradually increased during the proliferation of myoblasts. On the contrary, the m^6^A modification of total RNA gradually decreased (Figure 1C), roughly suggesting the demethylase function FTO plays on mRNA. 

To explore the effect of FTO on myoblast proliferation, siRNA targeting *FTO* mRNA (siFTO) was transfected into MuSCs cells and successfully knocked down *FTO* transcripts by ~0.6 fold (*p* < 0.01) (Figure 1D), while significantly up-regulating the m^6^A modification level of total RNA (*p* < 0.05) (Figure 1E). Interference with FTO only decreased mRNA levels of itself, but failed to change the expression of most methyltransferases, demethylases, and methyl-reading proteins (Figure 1F). In addition, the deficiency of FTO significantly decreased proliferation marker gene *mki67* (*p* < 0.05), but mRNA levels of *PAX7* and *PCNA* changed insignificantly (*p* > 0.05, Figure 1G). The WB experiment showed that the knockdown of FTO significantly reduced the protein of FTO and PAX7 by ~0.4 fold (*p* < 0.01 or 0.05) (Figure 1H). Moreover, the number of newly formed cells measured by the CCK8 (OD value at 450 nm) was lower in the siFTO group than in the control group at 24 h, 48 h, and 72 h and reached a significant level at 48 h (*p* < 0.01) (Figure 1I). On the contrary, overexpressed FTO resulted in more new cells than the control group (Figure S1). Consistently, compared with the control, FTO knockdown dramatically decreased the number of EdU-positive cells (*p* < 0.01) (Figure 1J). 

These results indicate that FTO promotes muscle cell proliferation.

### 2.2. DAG1 Is Targeted by FTO and Inhibits Cell Proliferation

**Figure 2:**
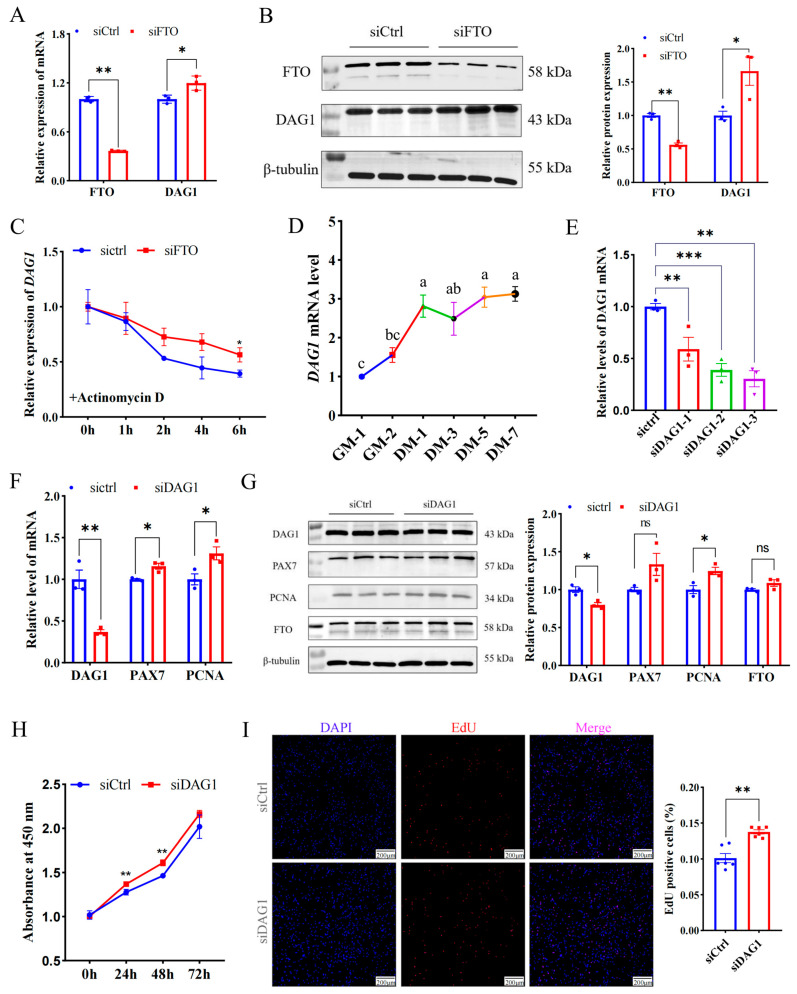
The FTO-targeted gene DAG1 inhibits cell proliferation. (**A**) *DAG1* mRNA increased by FTO knockdown. (**B**) DAG1 protein elevated by inhibiting FTO. (**C**) *DAG1* mRNA stability affected by FTO knockdown. (**D**) The profile of DAG1 in cell proliferation and differentiation. (**E**) siRNA targeting *DAG1* knockdown on mRNA expression. (**F**) mRNA changes in cell proliferation marker genes in cells deficiency of DAG1. (**G**) Effect of DAG1 knockdown on protein of cell proliferation marker genes. (**H**) Viability of cells tested by CCK8. (**I**) EdU staining cells altered by siDAG1. Scale bar: 200 μm. Results are represented as the mean ± SEM, * *p* < 0.05, ** *p* < 0.01, *** *p* < 0.001, and ns indicates no significance. In the picture marked with lower case letters, means shared at least one letter indicate no significance (*p* > 0.05), and on the contrary, no common letters indicate a significant difference (*p* < 0.05).

To explore the regulatory mechanism of FTO in promoting muscle cell proliferation, the potential targets of FTO were further screened using previously published data, and it was found that dystroglycan 1 (*DAG1*) is the gene most affected by it (Figure S2) [13]. In addition, several studies have unveiled that DAG1 regulates cell proliferation [14,15,16,17]. Here, we found interfering FTO significantly elevated DAG1 mRNA (*p* < 0.05) (Figure 2A) and protein (*p* < 0.05) (Figure 2B). Since m^6^A closely regulates mRNA stability [10], we assessed whether FTO stabilizes *DAG1* mRNA. siFTO-transfected cells were treated with actinomycin D for 0 h, 1 h, 2 h, 4 h, and 6 h. The results showed that compared with the control, a deficiency of FTO significantly increased the stability of *DAG1* mRNA (*p* < 0.05) (Figure 2C). These results suggest that FTO inhibited the expression of *DAG1* mRNA during myoblast proliferation by affecting its stability. 

Furthermore, *DAG1* mRNA expression increased significantly from the proliferative stage to the first day of differentiation, and then slowly decreased and then increased. (Figure 2D). Meanwhile, the successful knockdown of *DAG1* using three siRNAs (*p* < 0.01) (Figure 2E) significantly promoted mRNA levels of the proliferation marker genes *PAX7* and *PCNA* (*p* < 0.05) (Figure 2F). The WB results implied that a deficiency of DAG1 increased the protein of PCNA (*p* < 0.05) and PAX7 (*p* = 0.08), without a significant change in FTO (Figure 2G). The number of new cells tested by CCK8 in the siDAG1 group was higher than that in the control group at 24 h, 48 h, and 72 h (Figure 2H). Expectedly, overexpressing DAG1 decreased the cell number at 24 h, 48 h, and 72 h and reached a significant level at 72 h (*p* < 0.01) (Figure S3). Additionally, the knockdown of DAG1 significantly increased EdU-positive cells (*p* < 0.01) (Figure 2I). These results suggest that FTO negatively regulates DAG1, a suppressor of myogenic proliferation.

### 2.3. FTO Regulates DAG1 in an m^6^A-Dependent Manner

**Figure 3:**
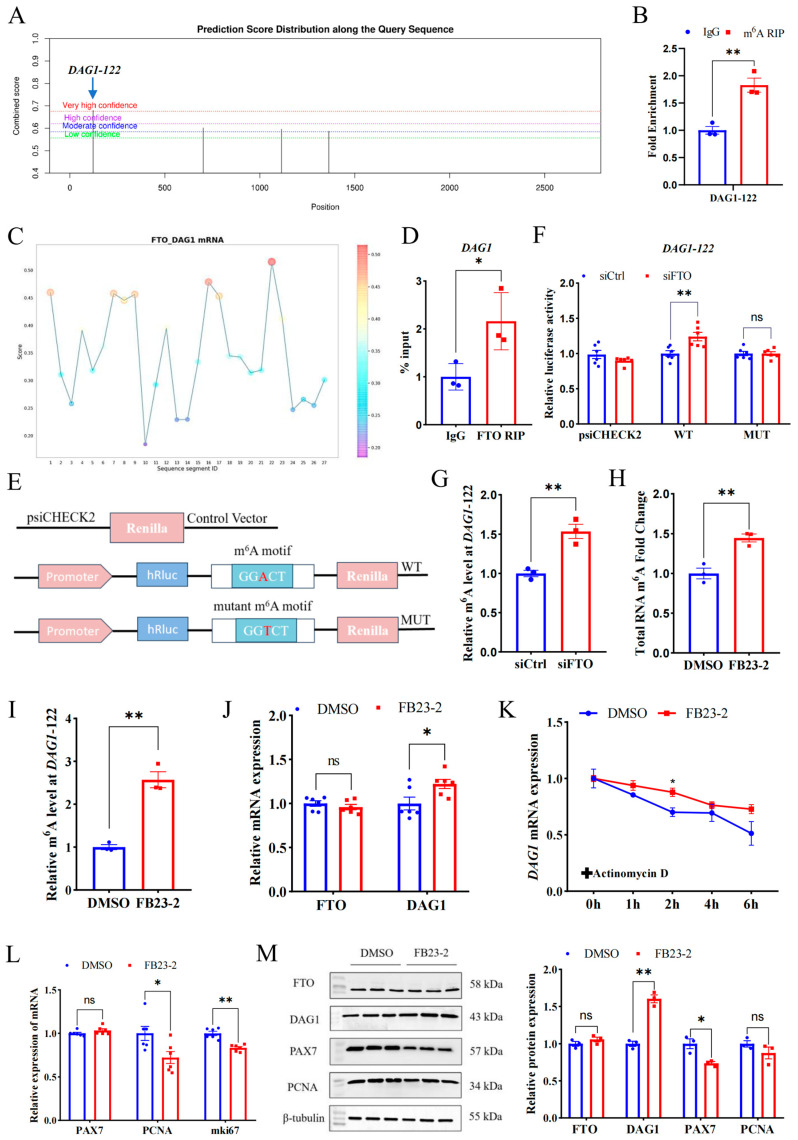
FTO regulates DAG1 and other proliferation genes in an m^6^A-dependent manner. (**A**) The m^6^A modification sites on *DAG1* mRNA predicted using the SRAMP. (**B**) The m^6^A modification of *DAG1* mRNA verified by MeRIP-qPCR. (**C**) The FTO binding sites on *DAG1* mRNA predicted by RBPsuite. (**D**) FTO binding on *DAG1* mRNA verified by RIP-qPCR. (**E**) The wild-type (WT) and mutant (MUT) m^6^A motif dual luciferase reporter vectors. (**F**) Effect of interfering FTO on luciferase activity in m^6^A-modified fragments of *DAG1* mRNA. (**G**) DAG1 m^6^A modification levels affected by interfering FTO. (**H**) Changes in total RNA m^6^A modification of cells treated by FB23-2. (**I**) MeRIP-qPCR of DAG1-122 after FB23-2 treatment. (**J**) FTO and *DAG1* mRNA altered by FB23-2. (**K**) Effect of FB23-2 on *DAG1* stability. (**L**) mRNA levels of cell proliferation marker genes changed by FB23-2. (**M**) Protein of proliferation marker genes influenced by FB23-2. Results are represented as the mean ± SEM, * *p* < 0.05, ** *p* < 0.01, and ns indicates no significance.

To investigate the mechanism underpinning FTO’s regulation on DAG1, we employed SRAMP, an online mammalian m^6^A modification website, to screen the m^6^A sites in the full sequence of the *DAG1* gene. The top loci with the highest confidence (DAG1-122, chr22) were selected for verification (Figure 3A). Using the specific primers targeting the site and m^6^A MeRIP samples performed with goat LD muscles, we verified that anti-m^6^A was highly enriched at DAG1-122 sites compared with anti-IgG (Figure 3B). Further, we used the online website RBPsuite and found that FTO potentially bound to *DAG1* mRNA, with high confidence at DAG1-122 sites (Figure 3C). Moreover, the results from FTO-RIP performed on the LD confirmed the binding of FTO to *DAG1* transcripts (Figure 3D).

A dual luciferase reporter assay was performed to further determine the active sites of FTO on *DAG1* mRNA. Wild and mutant types of DAG1-122 were constructed into the psiCHECK2 vector (Figure 3E) and co-transfected with siCtrl and siFTO. The results showed that siFTO significantly increased the luciferase activity of the DAG1-122 wild-type (*p* < 0.01), and site mutation abolished this change (Figure 3F). Moreover, the results of the MeRIP-qPCR showed that the m^6^A modification level of DAG1-122 was significantly increased after siFTO (*p* < 0.05) (Figure 3G). These results indicate that DAG1-122 plays a critical role in FTO’s regulation on DAG1.

In addition, FB23-2 was used to selectively inhibit FTO demethylase activity. Compared with the control DMSO, FB23-2 treatment failed to significantly change the FTO mRNA and protein levels (Figure 3J,M). Nevertheless, the m^6^A levels of total RNA and the DAG1-122 site were dramatically upregulated (*p* < 0.01) in the FB23-2 treatment group (Figure 3H,I). Notably, FB23-2 treatment significantly increased DAG1 at both the mRNA and protein levels (*p* < 0.05 or *p* < 0.01) (Figure 3J,M). Furthermore, FB23-2-conducted cells were treated with actinomycin D for 0 h, 1 h, 2 h, 4 h, and 6 h. The results showed that FB23-2 increased the stability of *DAG1* mRNA (Figure 3K). Moreover, the addition of FB23-2 dramatically reduced transcripts of *PCNA* and *mki67* (*p* < 0.05 or *p* < 0.01) (Figure 3L) and the PAX7 protein (*p* < 0.05) (Figure 3M).

These results suggest that FTO suppresses m^6^A’s modification of transcripts including DAG1 mRNA at DAG1-122 dependent on their demethylase activity and further affects the myogenic proliferation of MuSCs.

### 2.4. IGF2BP1 Stabilizes DAG1 mRNA through Recognizing Its m^6^A Modification

**Figure 4:**
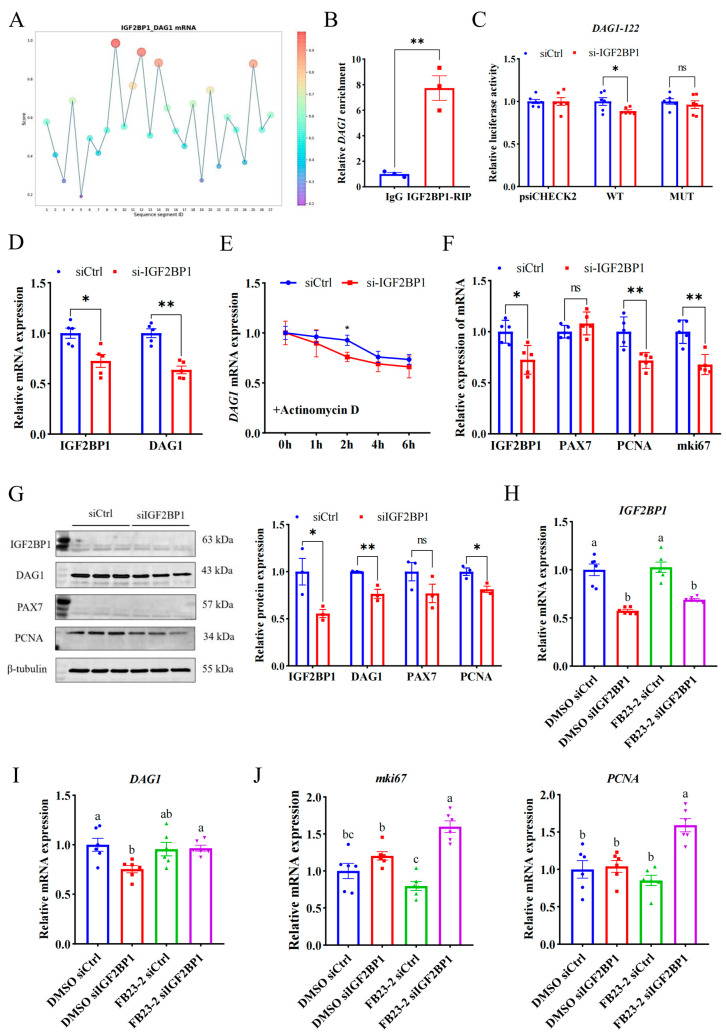
IGF2BP1 stabilizes *DAG1* mRNA through recognizing its m^6^A modification. (**A**) The DAG1-IGF2BP1 interaction sites predicted by RBPsuite. (**B**) *DAG1* mRNA enriched by IGF2BP1 protein. (**C**) Luciferase activity of DAG1-122 altered by interfering IGF2BP1. (**D**) IGF2BP1 and *DAG1* mRNA altered by interfering IGF2BP1. (**E**) *DAG1* mRNA stability caused by knockdown of IGF2BP1. (**F**) mRNA profiles of cell proliferation marker genes altered by deficiency of IGF2BP1. (**G**) Protein of cell proliferation marker genes affected by interfering IGF2BP1. (**H**) Expression of IGF2BP1 transcripts in cells treated with FB23-2 combined with siIGF2BP1. (**I**) *DAG1* mRNA, (**J**) *mki67* mRNA, and *PCNA* mRNA in cells cotransfected with FB23-2 and siIGF2BP1. Results are represented as the mean ± SEM, * *p* < 0.05, ** *p* < 0.01, and ns indicates no significance. Means with totally different lowercase letters indicate *p* < 0.05.

A reading protein is critical for recognizing the m^6^A modification site and downstream function, so it is important to explore the reading protein that recognizes the m^6^A modification site of *DAG1* mRNA. Since the m^6^A modification promotes *DAG1* mRNA expression, and IGF2BP1 is a typical reading protein that promotes mRNA stability [18,19,20], we used an online website and found that the IGF2BP1 potentially binds to *DAG1* mRNA at high confidence sites (DAG1-122) (Figure 4A). And the RIP assay confirmed that *DAG1* mRNA was significantly enriched by the IGF2BP1 antibody (Figure 4B). Furthermore, compared with the control, interference with IGF2BP1 significantly reduced the wild-type luciferase activity of DAG1-122 (*p* < 0.05), without changing after the mutation (Figure 4C). In addition, both mRNA and proteins of DAG1 were dramatically reduced after interfering with IGF2BP1 (*p* < 0.01) (Figure 4D,G). These results show that IGF2BP1 acts on the DAG1-122 locus and promotes DAG1 expression. 

To detect the effect of IGF2BP1 on DAG1 stability, siIGF2BP1-transfected proliferation cells were treated with actinomycin D for 0 h, 1 h, 2 h, 4 h, and 6 h. The results showed that compared with the control, the stability of *DAG1* mRNA was significantly reduced by a deficiency of IGF2BP1 and reached a significant level at 2 h (*p* < 0.05) (Figure 4E), suggesting that the recognition and binding of IGF2BP1 on DAG1-122 affected the stability of *DAG1* mRNA and promoted its expression during myoblast proliferation. In addition, the mRNA levels of *PCNA* and *mki67* were significantly reduced after siIGF2BP1 (*p* < 0.01) (Figure 4F), and the level of PCNA protein was significantly reduced (*p* < 0.05) (Figure 4G), indicating that IGF2BP1 promotes the proliferation of muscle cells.

In order to explore whether the regulation of IGF2BP1 on DAG1 depends on the demethylase activity of FTO, we co-transfected cells with siIGF2BP1 and FB23-2 to conduct a salvage test. The results showed that FB23-2 solely failed to affected IGF2BP1 levels, while the levels of *IGF2BP1* mRNA were significantly decreased by interfering itself (*p* < 0.05) (Figure 3H). Moreover, *DAG1* mRNA was downregulated by a deficiency of IGF2BP1 when FB23-2 was absent (*p* < 0.05), and this decrease was successfully restored by adding FB23-2 (*p* < 0.05) (Figure 3I). Additionally, the expression of *mki67* and *PCNA* was greatly elevated by the cotransfection of siIGF2BP1 and FB23-2 (*p* < 0.05), though FB23-2 solely tended to downregulate them (Figure 3J). These results suggest that IGF2BP1 positively regulates DAG1 expression through m^6^A modification, subsequently affecting myoblast proliferation.

During the proliferation of goat MuSCs, the demethylase FTO reduces the m^6^A methylation level of *DAG1* mRNA, which results in fewer recognition sites of IGF2BP1 and a consequently decreased DAG1 level and eventually promotes the proliferation of muscle cells. On the contrary, the knockdown of FTO or inhibition of its demethylation effect by adding FB23-2 increases the m^6^A methylation level of *DAG1* mRNA and the recognition of IGF2BP1 on the m^6^A site, promoting its expression, thereby inhibiting the proliferation of muscle cells.

## 3. Discussion

The regulatory network of muscle development is complex, and currently, most research focuses on various myogenic regulatory factors and non-coding RNA [21]. However, the post-transcriptional regulatory mechanisms affecting skeletal muscle development remain largely unknown. m^6^A modification is a methylation modification that occurs on the sixth nitrogen atom of RNA adenine and affects mRNA nucleation [18], mRNA splicing [19], stability [20], and other biological processes. Due to the development of m^6^A high-throughput sequencing technology, many studies have shown a correlation between m^6^A modification and skeletal muscle [22,23]. 

MeRIP-seq and RNA-seq combined analysis and comparison of m^6^A mRNA in yaks at different developmental stages showed that the gene regulating cell differentiation and muscle development was continuously expressed, and the abundance of m^6^A was negatively correlated with the gene expression level [24]. Whole-transcriptome studies revealed that m^6^A has a regulatory effect on embryonic muscle development in Wenchang chickens [25], Dingan goose [26], and ducks [27,28]. The m^6^A-seq analysis of skeletal muscle of wild boar, Landrace, and Rong chang pig (obese breed) showed that the m^6^A modification pattern had obvious differences among breeds [23]. In this study, FTO transcripts was enriched in goat skeletal muscles. Moreover, m^6^A modification levels of total RNAs exhibited two waves of decrease during myogenic development of MuSCs from the proliferative stage to the pre-differentiation stage (GM1 to DM1) and during differentiation stage (DM3 to DM7). This indicates that m^6^A modification is key in regulating myogenic development in goats.

Previous studies have shown that FTO relies on m^6^A modification to regulate the expression of NFATC1 to maintain the formation of slow muscle fibers in mice [29]. To further regulate the cell cycle process, the cyclin CCND1 is demethylated [30]. Previous studies have shown that the knockdown of both methyltransferase FTO and demethylase METTL3 promotes bovine myoblast proliferation, suggesting that m^6^A modifiers regulate skeletal muscle development with a complex and contradictory network of regulatory systems [31]. In addition, m^6^A modification-related enzymes are also regulated by other genes and small-molecule compounds [32,33,34,35,36]. It was reported that FTO knockdown significantly decreased the expression of cyclin CCND1 and inhibited the proliferation of goat myoblasts [13]. In this study, we came to a similar conclusion that FTO knockdown inhibited myocyte proliferation. In addition, the proliferation process of myoblasts is impaired when the expression of anti-muscular dystrophin is lost [37]. We found that decreased FTO expression level can promote the expression of DAG1, and DAG1 knockdown can promote myoblast proliferation.

In 2019, a study reported the discovery of a compound, FB23-2, with high activity and selectivity against FTO [38]. Subsequently, the compound was used to study cancer [39,40], bone development [41,42], and muscle cell proliferation [13]. In this study, the dual luciferin report test of m^6^A modification site mutation (DAG1-122) found that this site is a key site for FTO function. The agreement between the addition of FB23-2 to MuSCs and the interference with FTO strongly suggests that the regulation of DAG1 by FTO in proliferative MuSCs depends on m^6^A modification.

m^6^A modification requires reading proteins to recognize m^6^A and regulate mRNA processing [43]. m^6^A modification promotes *DAG1* mRNA expression, so the IGF2BPS family that promotes mRNA stability is selected. IGF2BP3 has been reported to promote the human myocardial regeneration process by promoting *MMP3* mRNA stability through m^6^A modification [44]. The IGF2BP1 protein can enhance its stability and facilitate translation by recognizing RNA N6-methyladenosine [11]. IGF2BP1 was also found to target many myogenic genes in skeletal muscle, such as MYH2 and MyoD, suggesting that IGF2BP1 may regulate skeletal muscle development by binding to target genes that undergo m^6^A modification [45]. Here, we found the binding of IGF2BP1 and *DAG1* mRNA through the RIP test, and IGF2BP1 recognized DAG1 m^6^A modification sites and increased mRNA expression, which affected the proliferation of goat muscle cells.

As part of epigenetic modification, m^6^A modification regulates a variety of developmental processes. In studies of FTO catalytic substrates, they were initially reported to catalyze the demethylation of 3-methylthymine (3-meT) [46] and 3-methyluracil (3-meU) [47] in single-stranded DNA, subsequently catalyzing N^6^-methyladenosine (m^6^A) [8] as the first RNA demethylase. The second base in many mRNAs can be methylated by 2′-O-methylation [48], with a portion of the base forming m^6^A_m_ from m^6^A [49]. N^6^,2′-O-dimethyladenosine (m^6^A_m_) is also a reversible modification that affects the fate of cellular mRNA [50]. FTO preferentially demethylates m^6^Am rather than m^6^A and reduces the stability of m^6^A_m_ mRNA. However, under certain conditions, the overexpression efficiency of FTO is high, and the level of m^6^A in mRNA will be reduced [51]. Our findings are consistent with a slight increase in total m^6^A levels in cell lines after FTO knockdown [8]. It is worth noting that the overall abundance of m^6^A_m_ is much lower than that of m^6^A. Most importantly, m^6^A modification has a strong correlation with muscle development, and there are currently no studies showing a relationship between m^6^A_m_ and muscle development, which may also be a direction for future research.

**Figure 5:**
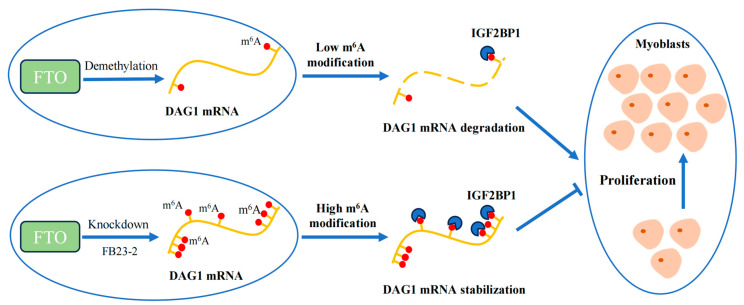
Proposed mechanism of FTO/IGF2BP1/DAG1 on myocyte proliferation.

Conclusively, our study shows that demethylase FTO affects the m^6^A modification of total RNA during muscle development. In addition, FTO regulates DAG1 levels and thus promotes muscle cell proliferation. Mechanistically, FTO demethylates the m^6^A modification of *DAG1* mRNA, which interrupts the m^6^A-related mRNA stability recognized by IGF2BP1, thereby promoting muscle cell proliferation (Figure 5).

## 4. Materials and Methods

### 4.1. Animal and Sample Collection

Goats were from the Chengdu Ma Goat breeding farm in Dayi County, Chengdu, China. Three healthy 1-month-old Chengdu Ma goats were humanely sacrificed. Their hearts, livers, spleen, lungs, kidneys, longissimus dorsi muscles, psoas major muscles, gastrocnemius, and semitendinosus muscles were sampled. The samples were quickly placed in liquid nitrogen for RNA extraction. 

### 4.2. Isolation and Identification of MuSCs

The longissimus dorsi muscles of the newborn Chengdu Ma goats were quickly collected and cleaned in PBS buffer. After removing fascia, adipocyte tissue, and blood vessels, the muscle was cut into pieces and digested with trypsin and collagenase I + II. When the tissue digestion was completed, 10% FBS was added to terminate digestion, and then, the tissue was mixed and slowly filtered with a 70 μm cell sieve. Filtrate was collected in a 50 mL centrifuge tube and centrifuged at 2000 rpm for 5 min, and the supernatant was discarded. After full cleaning with PBS, centrifuge it was centrifuged and the supernatant was discarded under the same conditions. Finally, 5 mL of 10% FBS resuspension cells was added, mixed, and cultured in an incubator (37 °C, 5% CO_2_) [51]. Moreover, the isolated cells were identified by staining with a PAX7 antibody (with a positive rate > 95%) and could successfully differentiate into myocytes and myotubes (MyHC-positive). These isolated cells were considered MuSCs and were used for subsequent experiments.

### 4.3. Cell Culture and Transfection

MuSCs were cultured in a growth medium containing 10% FBS (Gibco, Grand Island, NY, USA) and 1% penicillin–streptomycin (Invitrogen, Bohemia, NY, USA). When the cell density reached more than 85%, it was replaced with 2% equine serum (Gibco, Grand Island, NY, USA)—cultured in a differentiation medium to promote myogenic differentiation. Lipofectamine 3000 (Life Technologies, Waltham, MA, USA) was used in transfected plasmids.

### 4.4. Cell Proliferation Assay

An appropriate amount of 50 μM EdU medium was prepared, and on the second day after experimental treatment (transfection of interfering RNA) in MuSCs, 100 μL of 50 uM EdU medium was added to each well and incubated for 2 h, the medium was discarded, and the cells were cleaned with PBS 1–2 times, for 5 min each time. Follow-up cell fixation, apollo staining, and other steps were carried out according to the instructions for the Cell-Light™ EdU Apollo In Vitro Kit (RiboBio, Guangzhou, China). 

The cells were inoculated on 96-well plates and treated (transfected with interference RNA) after adhesion. After incubation for 24, 48, and 72 h in the incubator, 10 μL of CCK-8 solution was added to each well and incubated in the incubator for 2 h in the dark to detect the absorbance of 450 nm. The CCK8 experiment used Cell Counting Kit-8 (Beyotime, Shanghai, China).

### 4.5. Target Gene Screening Methods

Genes related to muscle development were screened according to qval < 0.05 and 0 < |log_2_.FC| < 2. As shown in the figure below, DAG1 was the gene most affected by FTO in proliferation cells, and the level of m^6^A was high. Therefore, DAG1 was finally selected as the target of FTO for further exploration.

### 4.6. Interfering RNA and Plasmid Construction

The siRNA used in this experiment was designed and synthesized by Ruibo Biology (RiboBio, Guangzhou, China), and the siRNA information is shown in Table S1. The complete CDS of the FTO (NC_030825.1) gene was amplified and inserted into the pEGFP-N1 (Promega, Madison, WI, USA) vector using a homologous recombinant cloning kit (Vazyme, Nanjing, China) to construct the overexpressed plasmid. The DAG1 (NC_030829.1) overexpression vector was synthesized by Qingke (Beijing, China).

### 4.7. Total RNA Extraction and qPCR

RNAiso Plus (Takara, Dalian, China) was used to extract RNA from cells and tissues. cDNA was obtained using a PrimeScript™ RT kit (Takara, Dalian, China). In addition, SYBR premix Ex TaqTM II (Takara, Dalian, China) was employed to quantify gene expression by qPCR. The 2^−ΔΔCt^ methods were used to normalize gene levels with GAPDH or β-actin as the internal control. All primers are shown in Table S2.

### 4.8. Immunofluorescence

MuSC was washed thrice with PBS (5 min each time) and fixed with 4% paraformaldehyde. The cells were permeated with 0.5% TritonX-100. The cells were then incubated with the primary antibody (1:120, 4 °C, 12 h) and the secondary antibody (1:120, 37 °C, 2 h). Finally, the calculation was performed using ImageJ software (Version 1.54j). Antibodies are as follows: anti-Pax7 (1:200, abs124153, absin, Shanghai, China), anti-MyHC (1:300, sc-378137, Santa Cruz, CA, USA), Cy3 Goat Anti-Mouse IgG (1:200, AS008, ABclonal, Wuhan, China), and FITC Goat Anti-Rabbit IgG (AS011, 1:200, ABclonal, Wuhan, China).

### 4.9. Western Blot (WB) Analysis

About 20 micrograms of protein was separated using 8%–12% SDS-PAGE gel, transferred to activated (PVDF) membrane (Millipore, MA, USA), cleaned with TBST solution, and sealed with 5% skim milk at 37 °C for 2 h. After TBST cleaning, the membrane was incubated with the required primary antibody at 4 °C overnight. After TBST cleaning, the corresponding secondary antibody was incubated at 37 °C for 1.5 h after dilution. TBST was cleaned in sequence with TBS and strip visualization was performed using Ge1DocTM XR & ChemiDocTM XRS (Bio-Rad, Hercules, CA, USA).

The total protein extraction kit (Beibo Biology, Shanghai, China) was used to extract protein, and the BCA protein quantification kit (Beibo, Shanghai, China) was used for protein quantification. WB antibodies mainly include anti-β-tubulin (1:1000, 250007, ZENBIO, Chengdu, China), anti-MyHC (1:1000, sc-378137, Santa Cruz, Dallas, TX, USA), anti-FTO (1:1000, bs-7056R, Bioss, Woburn, MA, USA), anti-DAG1 (1:1000, A10076, ABclonal, Woburn, MA, USA), anti-PAX7 (1:200, sc-81975, Santa Cruz, CA, USA), anti-Mouse IgG (1:5000, 511103, ZENBIO, Chengdu, China), and anti-Rabbit IgG (AS014, 1:5000, ABclonal, Wuhan, China).

### 4.10. Luciferase Reporter Assays

We downloaded the mature mRNA sequence of goat DAG1 (NC_030829.1) from the NCBI official website. Wild-type (WT) and m^6^A motif mutants (modification sites A to T mutation, MUT) from the DAG1-122 regions were inserted into the psiCHECK-2 vector. siCtrl and siFTO were transfected into MuSCs, and subsequently, psi-CHRCK2, WT, and MUT vectors were transfected. Luciferase activity was quantified using the dual luciferase reporter gene assay kit (Transgen, Beijing, China). The MUT vector was synthesized by Cytology (Qingke, Beijing, China). These luciferase vector sequence are shown in Table S3.

### 4.11. Total m^6^A Modification Level Analysis

RNAiso Plus was used to isolate total RNA from MuSCs at proliferative stages (GM-1, GM-2) and differentiation stages (DM-1, DM-3, DM-5 and DM-7). The global m^6^A methylation level of RNA was detected using the m^6^A RNA methylation quantitative kit (Epigentek, Farmingdale, NY, USA).

### 4.12. RNA Immunoprecipitation (RIP) Assay

The MuSC was collected and cleaved for RIP assays to evaluate the binding capacity of proteins and DAG1 mRNA. Subsequently, RIP was tested using the Magna RIP™ RNA-binding protein immunoprecipitation kit (Sigma-Aldrich, St. Louis, MO, USA). The antibodies used in RIP assays include FTO (1:1000, 27226-1-AP, Proteintech, Rosemont, IL, USA), IGF2BP1 (1:1000, EPR18797, abcam, Cambridge, UK), and IgG (Sigma-Aldrich, St. Louis, MO, USA).

### 4.13. RNA Stability Assays

The cells were treated according to the experimental requirements (transfected with siFTO or siIGF2BP1 or added with FB23-2 on the second day of muscle cell proliferation), and actinomycin D was added at the appropriate time (48 h after transfection and 72 h after the addition of FB23-2). The cells were collected 0, 1, 2, 4, and 6 h later, and RNA was extracted for subsequent experiments.

### 4.14. Methylated RNA Immunoprecipitation (MeRIP)

First, RNA was extracted from LD tissues of Chengdu Ma goats and cells were transfected with siFTO/FB23-2 on the second day of proliferation; then, we enriched the m^6^A-modified RNA fragments in cells with antibodies that exclusively recognized m^6^A modification through the Magna MeRIP™ m^6^A Kit (Sigma-Aldrich, St. Louis, MI, USA). The enriched RNA was purified using the RNeasy mini kit (Qiagen, Hilden, Germany). Finally, the m^6^A level of DAG1 mRNA was quantified by qPCR.

### 4.15. Online Prediction of m^6^A Modification Sites and RBP Binding

To predict m^6^A modification sites on the DAG1 transcripts, the mature mRNA sequence was put into the prediction box using the online tool SRAMP with mature mRNA mode (http://www.cuilab.cn/sramp, accessed online 5 May 2023).

We employed RBPsuite (http://www.csbio.sjtu.edu.cn/bioinf/RBPsuite/, accessed online 21 June 2023) to explore the potential interaction between proteins and transcripts. We chose the human species, linear RNA type, and general protein model and then filled in the RNA sequence as required. Subsequently, we obtained a map of the protein distribution on RNA.

### 4.16. Statistical Analysis

At least three independent biological replicates were performed for each experiment or treatment. Data were analyzed using GraphPad Prism version 9.0. An unpaired two-tailed Student’s *t*-test was employed to compare the means difference between the two groups when the homogeneity of variance met Levene’s test. The one-way or two-way ordinary ANOVA with Tukey’s correction for multiple comparisons was performed to analyze three or more means with equal variances. All results were presented as mean ± SEM. Statistical significance was denoted as * *p* < 0.05, ** *p* < 0.01, and *** *p* < 0.001.

## Data Availability

The data from the current study are exhibited in the manuscript and Supplementary Materials.

## Supplementary Materials

The following supporting information can be downloaded at: https://www.mdpi.com/article/10.3390/ijms25189804/s1.
