# Supplementary material for: Fat Mass- and Obesity-Associated Protein (FTO) Promotes the Proliferation of Goat Skeletal Muscle Satellite Cells by Stabilizing DAG1 mRNA in an IGF2BP1-Related m6A Manner
Source: Int J Mol Sci. 2024 Sep 11;25(18):9804. doi: 10.3390/ijms25189804 (PMC11432635; doi:10.3390/ijms25189804)
Supplement: Supplementary file 1 [file ijms-25-09804-s001.zip › Figure S2.pdf]

S2

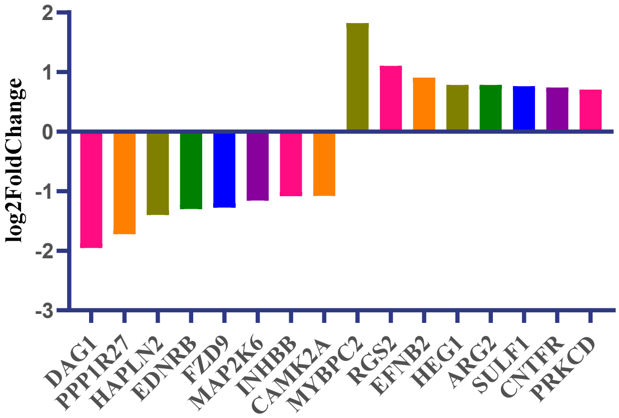

Figure S2. Expression patterns of genes interfered by FTO. Genes related to muscle development were screened according to qval < 0.05 and 0 <|$\log_2$.FC|< 2.
